# Subjective Outcome Evaluation of the Project P.A.T.H.S. (Extension Phase) Based on the Perspective of Program Implementer

**DOI:** 10.1100/2012/589257

**Published:** 2012-08-02

**Authors:** Daniel T. L. Shek, Lu Yu

**Affiliations:** ^1^Department of Applied Social Sciences, The Hong Kong Polytechnic University, Hong Kong; ^2^Public Policy Research Institute, The Hong Kong Polytechnic University, Hong Kong; ^3^Department of Social Work, East China Normal University, Shanghai 200241, China; ^4^Kiang Wu Nursing College of Macau, Macau; ^5^Division of Adolescent Medicine, Department of Pediatrics, Kentucky Children's Hospital, University of Kentucky College of Medicine, Lexington, KY 40506, USA

## Abstract

A total of 231 schools participated in the Project P.A.T.H.S. in 2009/2010 school year. After completion of the Tier 1 Program, subjective outcome evaluation data were collected from 3,259 program implementers. Based on the consolidated data with schools as units, results showed that participants had positive perceptions of the program, implementers, and benefits of the program. More than four-fifth of the implementers regarded the program as helpful to the program participants. Multiple regression analyses revealed that perceived qualities of the program and the program implementers predicted perceived effectiveness of the program. Similar to previous studies, compared to implementers' perception about their performance, the perceived program content appeared to be a stronger predictor of program success. The present study provides additional support for the effectiveness of the Tier 1 Program of the Project P.A.T.H.S. in Hong Kong.

## 1. Introduction

How to prevent adolescent risk behavior, such as delinquency, drug abuse, unprotected sexual behavior, and school failure, has been a challenging issue for psychologists, educators, policy makers, and other helping professionals [1–3]. In recent years, the research paradigm that different adolescent risk behaviors are treated as separate and independent problems is changing. Instead, emphasis has been put on the interconnections among various risk behaviors and their shared risk, protective, and facilitative factors. Both theoretical models and empirical studies have supported one common predictor of a wide range of risk behaviors in youth—positive youth development or youth developmental assets [4]. Accordingly, numerous youth programs have been developed with a focus on promoting the development of core competences and adaptive features of adolescents, which can be generally subsumed under the category of positive youth development approach [5–7].

The approach of positive youth development has been widely adopted in designing programs for adolescents in the west [8]. However, such programs are rarely developed and carried out in Asian countries, especially different Chinese communities [9]. In view of this situation, Shek and researchers from five universities in Hong Kong developed a large-scale positive youth development program entitled the Project P.A.T.H.S. (Positive Adolescent Training through Holistic Social Programmes) to promote healthy development in Hong Kong adolescents and to prevent various youth risk behaviors [10, 11]. Funded by the Hong Kong Jockey Club Trust Charities since 2005 (with funding of HK$400 million in the initial phase and HK$350 million in the extension phase), the project has been implemented in about half of the secondary schools in Hong Kong for consecutively seven years. There are two tiers of program in the project. Tier 1 Program is a universal positive youth development program provided for secondary 1 to 3 students in Hong Kong. Tier 2 Program takes a selective approach which aims at around one-fifth of the Tier 1 Program participants who have greater psychosocial needs.

As the Project P.A.T.H.S. has been implemented in a large scale in Hong Kong adolescents, one important question that must be asked is how effective the project is. To answer this question, systematic evaluation of the program is necessary. Since the launch of the program, numerous evaluation studies have been carried out, with the use of a variety of evaluative strategies, including objective outcome evaluation, subjective outcome evaluation, focus group interview, case studies, direct observation, and a longitudinal randomized group controlled trial [12, 13]. Findings based on these evaluation studies in the past seven years have generally shown positive program effects of the Project P.A.T.H.S. in promoting different competences and developmental assets and preventing various risk behaviors in the program participants [14–17]. For example, based on eight waves of data collected in five consecutive years, Shek and colleagues reported that students who had participated in the Project P.A.T.H.S. showed better developmental outcomes than did students in a randomized controlled group, in terms of both positive youth development indicators (e.g., resilience, moral competence, and prosocial involvement) and different risk behaviors such as substance abuse and delinquent behaviors [18, 19].

While objective outcome evaluation, particularly randomized controlled trial, is considered the “gold” standard for the assessment of program effectiveness, subjective outcome evaluation has several unique advantages in program evaluation [20–22]. First, as compared to objective outcome evaluation, subjective outcome evaluation provides a way to find out different stakeholders' opinions and subjective experiences of the program. Second, subjective outcome evaluation offers immediate and important information about the implementation of a program before its effects on objective indicators can be observed. Third, subjective outcome evaluation is a more cost-effective evaluative method than objective outcome evaluation. Fourth, subjective outcome evaluation by program implementers contains valuable message about problems and difficulties encountered in program implementation which contribute to the improvement of the program in the future. In evaluating the Project P.A.T.H.S., subjective outcome evaluation was conducted in both program participants and program implementers to obtain a comprehensive picture about different stakeholder's views towards the project [23].

Although very encouraging evaluation findings have been reported for the initial phase of the project, it is important to know whether similar positive findings could be found for the extension phase. Against this background, subjective outcome evaluation findings based on the perspectives of program workers who implemented the Tier 1 Program in the 2009/2010 school year were reported in this paper. In addition, instructors' perceptions about the program, their own performance, and the effectiveness of the project were contrasted among different grade levels to learn about whether program workers at different grades have different views about the program. Previous findings suggested that instructors who taught the curriculum in the lower forms had more positive perceptions than did instructors teaching the program in the higher forms. As such, it was hypothesized that similar pattern regarding the grade effect on program implementers' subjective evaluation would also be observed in the present sample. Besides, the relationships among program implementers' views towards the program, perceptions about the instructor, and the overall effectiveness of the program were examined to gain a further understanding of critical factors that influence perceived program effectiveness by program workers. Based on prior findings, it was hypothesized that program implementers' perceived program quality and their own performance would significantly predict their subjective evaluation on program effectiveness.

## 2. Methods

### 2.1. Participants and Procedures

A total of 231 schools joined the Project P.A.T.H.S. in the fourth year of the full implementation phase in the 2009/2010 school year (i.e., the first year in the extension phase), with 219, 185, and 173 schools in secondary 1, secondary 2 and secondary 3 levels, respectively. The mean number of students per school was 154.36 (ranged from 6 to 240 students), with an average of 4.50 classes per school (ranged from 1 to 12 classes). Among them, 32.24% of the respondent schools adopted the full program (i.e., 20-hour program involving 40 units) whereas 67.76% of the respondent schools adopted the core program (i.e., 10-hour program involving 20 units). The mean number of sessions used to implement the program was 28.54 (ranged from 2 to 48 sessions). While 47.31% of the respondent schools incorporated the program into the formal curriculum (e.g., Liberal Studies, Life Education), 52.69% used other modes (e.g., using form teacher's periods and other combinations) to implement the program. The mean numbers of social workers and teachers implementing the program per school per form were 1.71 (ranged from 0 to 7) and 5.11 (ranged from 0 to 27), respectively.

After the Tier 1 Program was completed, the implementers were invited to respond to a Subjective Outcome Evaluation Form (Form B) developed by the first author [24]. In the school year 2009-2010, a total of 3,259 questionnaires were completed. The data collection was conducted after the completion of the program. To facilitate the program evaluation, the Research Team developed an evaluation manual with standardized instructions for collecting the subjective outcome evaluation data [24]. In addition, adequate training was provided to the implementers during the 20-hour training workshops on how to collect and analyze the data collected by Form B.

### 2.2. Instruments

The Subjective Outcome Evaluation Form (Form B) was used in the present study, including the following parts.

- Program implementers' perceptions of the program, such as program objectives, design, classroom atmosphere, interaction among the students, and the respondents' participation during class (10 items).
- Program implementers' perceptions of their own practice, including their understanding of the course, teaching skills, professional attitude, involvement, and interaction with the students (10 items).
- Implementers' perceptions of the effectiveness of the program on students, such as promotion of different psychosocial competencies, resilience, and overall personal development (16 items).
- The extent to which the implementers would recommend the program to other students with similar needs (1 item).
- The extent to which the implementers would teach similar programs in future (1 item).
- The extent to which the program implementation has helped the implementers' professional growth (1 item).
- Things that the implementers obtained from the program (open-ended question).
- Things that the implementers appreciated most (open-ended question).
- Difficulties encountered (open-ended question).
- Areas that require improvement (open-ended question).

For the quantitative data, the program workers who collected the data were requested to input the data in an EXCEL file developed by the Research Team which would automatically compute the frequencies and percentages associated with the different ratings for an item. When the schools submitted the hard copy of the reports, they were also requested to submit the soft copy of the consolidated data sheets. After receiving the consolidated data by the funding body, the research team aggregated the data to “reconstruct” the overall profile based on the subjective outcome evaluation data. It should be noted that although both qualitative and quantitative data were collected, the present paper only focused on the quantitative reports. Qualitative findings are to be reported elsewhere.

### 2.3. Data Analysis

Percentage data were examined using descriptive statistics. A composite measure of each factor (i.e., perceived qualities of program content, perceived qualities of program implementers, and perceived program effectiveness) was created based on the total scores of each scale divided by the number of items. Pearson correlation analysis was used to examine if the program content and program implementers were related to the program effectiveness. To compare program implementers' evaluation across different grades, several one-way ANOVAs were conducted with the three subscale scores as the dependent variables and grade as the independent variable. Hierarchical linear regression analyses were further performed to examine the relationship between different aspects of implementers' evaluation about the project and the program effectiveness. All analyses were performed by using the Statistical Package for Social Sciences Version 17.0.

## 3. Results

The quantitative findings based on the closed-ended questions are presented in this paper. Several observations can be highlighted from the findings. First, the participants generally had positive perceptions of the program (Table 1), including clear objectives of the curriculum (94.80%), well-planned teaching activities (90.51%), and very pleasant classroom atmosphere (87.88%). Second, a high proportion of the implementers had positive evaluation of their performance (Table 2). For example, 98.18% of the implementers perceived that they were ready to help their students; 97.43% of the implementers expressed that they cared for the students; 96.35% believed that they had good professional attitudes. Third, as shown in Table 3, many implementers perceived that the program promoted the development of students, including their resilience (91.84%), social competence (93.73%), life reflections (91.61%), and overall development (93.16%). Fourth, 89.73% of the implementers would recommend the program to students with similar needs. Fifth, 83.36% of the implementers expressed that they would teach similar courses again in the future. Finally, 84.37% of the respondents indicated that the program had contributed to their professional development.

Reliability analysis with the schools as the unit of analyses showed that Form B was internally consistent (Table 4): 10 items related to the program (*α* = .95), 10 items related to the implementer (*α* = .94), 16 items related to the benefits (*α* = .98), and the overall 36 items measuring program effectiveness (*α* = .98). Results of correlation analyses showed that both program content (*r* = .79, *P* < .01) and program implementers (*r* = .65, *P* < .01) were strongly associated with program effectiveness.

To examine differences in the perceived variables (i.e., program content, program implementers, and program effectiveness) across grade levels, several one-way ANOVAs were performed with the perceived variables as dependent variables and grade level (i.e., secondary 1 to 3) as independent variable. Significant results were only found in program content, *F*_(2,574)_ = 3.77, *P* = .02. Post hoc analysis using Tukey's procedure with Bonferroni adjustment (i.e., *P* = .02) revealed that significant difference was found between secondary 1 (*M* = 4.47) and secondary 3 (*M* = 4.36) participants (*P* = .03), with the secondary 1 program perceived to be relatively more favorable than the Secondary 3 Program.

Multiple regression analyses were performed on both the whole sample and the responses of students in different grades separately.  Table 5 presents the findings. Overall, higher positive views towards the program and program implementers predicted higher perceived program effectiveness (*P* < .01). The prediction of program effectiveness was stronger for perceptions of program (*β* = .70) than for views towards implementers (*β* = .13). The model explained 63% of the variance toward the prediction of program effectiveness. For participants in different grades, the pattern of relationships and the amount of variance in program effectiveness explained by the two predictors were very similar. While views towards program content consistently predicted program effectiveness across grades, the relationship between views towards implementers and program effectiveness was only significant for the analyses based on the secondary 2 participants.

## 4. Discussion

The present study investigated the subjective outcome evaluation by program workers who implemented the Tier 1 Program of the Project P.A.T.H.S. in the 2009/2010 academic year. The findings showed that program implementers generally held positive views towards the program and the instructors and perceived the program as effective to promote healthy development of the participants. Program implementers' perceptions about the program and instructor significantly predicted their subjective evaluation about the program effectiveness, with views towards program content being a stronger predictor than views towards instructors. Moreover, these findings were held true for participants from different grade levels. There are three unique features of this study. First, the sample size was quite large. Actually, it is very rare to see such a large number of program implementers participated in outcome evaluation in the literature. Second, a validated measure of subjective outcome evaluation was used. Third, as there are few studies on the evaluation of positive youth development programs in general, particularly in Chinese people, the present study is an important addition to the literature.

Overall, more than 80% of the participated program implementers had positive evaluation about different aspects of the program content, including the good curriculum design, strong theoretical support, pleasant classroom atmosphere, and active participation of the students. In particular, more than 90% of the instructors agreed that the objectives of the curriculum were very clear and the activities were carefully planned. Explicit learning objectives with respect to the required skills and a variety of instructional activities to facilitate learning are two critical components in outcome-based education which embraces the notion that the learner is accountable for his or her own achievements and represents the most updated approach to nowadays education [25–28]. The present findings that these two items received the highest subjective evaluation from teachers suggest that the outcome-based approach has been well incorporated in the implementation of the Project P.A.T.H.S. Other opinions from teachers such as “there was much peer interaction amongst the students” and “on the whole students like this curriculum very much” provide further support for the successfulness of using this approach to deliver the Project P.A.T.H.S. in Hong Kong students.

Program implementers also viewed their own performance in teaching the program favorably, in terms of mastery and preparedness of the curriculum, teaching skills and attitudes towards the course and students, personal gains, interaction with students, and general evaluation of oneself as an instructor of the program. While self-fulfilling prophecy may explain the findings, it is noteworthy that this observation is consistent with previous findings that the students also perceived the instructors in a favorable light [12, 29], hence supporting the validity of the present finding.

With respect to the perceived effectiveness of the program, program implementers regarded the program as having promoted positive development in the participated students in multiple areas. For example, more than 90% of the instructors agreed that the project had enhanced students' bonding with others, resilience in adverse conditions, social competence, ability to make sensible and wise choices, and overall development. Students who attended the program were evaluated as having more life reflections and self-awareness. These findings are consistent with previous results based on other evaluation methods regarding the effectiveness of the Project P.A.T.H.S., such as the objective outcome evaluation and the subjective evaluation by students [29, 30].

Program implementers' subjective outcome evaluation was also compared among different grades. No significant grade differences were detected in program implementers' views about their own performance and perceived effectiveness of the program, which suggests that program implementers from different grade levels had similar favorable views towards the instructor and the program effectiveness. However, it was found that program content was evaluated more positively by secondary 1 implementers than by secondary 3 implementers. Similar findings were also noted in previous studies. While the curriculum designed for different grades has different content including various activities and topics for discussion, the basic framework of the course that consists of eight core positive youth development constructs is the same across grade. Therefore, the secondary 1 program may be perceived as more fresh and attractive to teachers than secondary 3 program. Besides, students in junior grade may also show more interests and better involvement in the course than senior students who attended the program since they entered to the secondary school. This finding provides some insights for the curriculum design in the future. Perhaps more novel units and topics especially suitable for senior secondary students could be developed and incorporated into the curriculum. Despite the grade difference, program implementers in the secondary 3 grade still reported favorable views towards the curriculum, with more than three fourths of the participants having positive evaluation about different aspects of the program content, which suggests that the curriculum is generally well received by the instructors.

Results of regression analyses suggest that for the whole sample of students, both perceived program and instructors significantly predicted the perceived effectiveness of the program, supporting the critical roles of program quality and implementers in program success. However, when data in different grades were analyzed separately, while program workers' subjective evaluation of the program quality consistently predicted perceived effectiveness of the program across grade, the effect of views about instructors' performance was only significant for secondary 2 participants. Apparently, program worker's evaluation about the program content appeared to be a stronger predictor than did their evaluation about instructors' performance. Similar findings were reported in Shek et al.'s paper [31]. While a variety of factors at different ecological levels were found to affect the implementation of a program, high program quality has always been considered the first requisite to the success of the program [32, 33]. Without a good design of the curriculum in the very beginning, it is impossible that the program will produce desirable outcomes in its participants, even with excellent program staff, highly-motived students, and supportive administrative environment. In fact, it is likely that quality of program and quality of implementers interactively affect the effectiveness of a program. For example, good curriculum content often increases the interests and motivation of instructors to teach the course [34], and thus the instructors may spend more time in preparation, show more passion in their teaching, and deliver the content in a more effective way. Therefore, when the effects of program content were controlled, the prediction of program instructors' performance on program effectiveness decreased. Another possibility is that this may be a statistical artifact as the range of scores for the evaluation of instructors was not wide. Future studies may focus on examining the interactive effects between program content and program implementers to identify more fundamental factors that determine program success. In addition, it is unclear why in the present study the evaluation of instructors only predicted program effectiveness for secondary 2 participants, but not for secondary 1 and 3 participants. This finding is inconsistent with previous report [31] and the literature in which the critical role of program implementers to program success is constantly highlighted [13, 21]. Obviously, replication study is needed. In particular, grade difference in the effect of program implementers' performance on program effectiveness should be further explored.

There are several limitations of the present study that should be acknowledged. First, the data were collected in a self-reported manner, which may be biased by the implementers' personal attitudes and perceptions towards the program. To reduce the potential bias, several measures were taken. First, program implementers responded to the questionnaire anonymously, and the confidentiality was repeatedly assured. Second, in the questionnaire, no threatening questions were asked that might elicit the respondents' feelings of role conflict and social desirability. Third, participants were encouraged to candidly report their negative views or feelings in the survey, and open-ended questions were provided for the teachers to record their suggestions on how to improve the program. Despite of these measures, the present findings should be interpreted with cautions, and evaluative studies that use other approaches, such as objective outcome evaluation based on developmental indicators, program participants' subjective evaluation, and process evaluation must be conducted for the purpose of triangulation. The second limitation of the present study is that only two general indicators of program quality and program implementers' performance were used to predict overall effectiveness of the project, which makes it impossible to identify specific aspects that are particularly important for program success. Besides, different factors may increase/decrease the program effects in different areas. For example, good performance of the teacher may have particular effects in strengthening students' bonding with teachers. Future studies may include different indicators of program content and implementers as well as program effectiveness in the prediction model. Thirdly, previous studies have revealed that school and organization characteristics influence program effectiveness and implementation quality [34–36]. These contextual factors should be considered in further research. Finally, as the present findings were “reconstructed” from the evaluation reports submitted by the agencies, the unit of analyses was schools, instead of individuals. Therefore, individual variations were lost in the process which may lower the power of statistical analyses. Despite of these limitations, the present study constitutes an important addition to the current literature about the effectiveness of the Project P.A.T.H.S. in promoting positive youth development in Hong Kong adolescents.

## Figures and Tables

**Table 1:** Summary of the program implementers' perception towards the program.

	Respondents with positive responses (options 4–6)							
	S1		S2		S3		Overall	
	*n*	%	*n*	%	*n*	%	*N*	%
(1) The objectives of the curriculum are very clear.	1214	95.37	979	94.86	887	93.96	3080	94.80
(2) The design of the curriculum is very good.	1125	88.37	873	84.68	803	85.06	2801	86.24
(3) The activities were carefully planned.	1160	91.19	924	89.71	854	90.47	2938	90.51
(4) The classroom atmosphere was very pleasant.	1156	90.95	897	87.17	796	84.50	2849	87.88
(5) There was much peer interaction amongst the students.	1127	88.74	875	84.87	776	82.73	2778	85.77
(6) Students participated actively during lessons (including discussions, sharing, games, etc.).	1121	88.06	866	84.00	755	80.15	2742	84.47
(7) The program has a strong and sound theoretical support.	1090	85.69	889	86.06	812	86.11	2791	85.93
(8) The teaching experience I encountered enhanced my interest in the course.	1051	82.76	826	80.19	751	79.64	2628	81.04
(9) Overall speaking, I have very positive evaluation of the program.	1071	84.26	839	81.30	756	80.08	2666	82.11
(10) On the whole, students like this curriculum very much.	1086	85.38	816	79.15	729	77.31	2631	81.05

*Note: *all items are on a 6-point Likert scale with 1 = strongly disagree, 2 = disagree, 3 = slightly disagree, 4 = slightly agree, 5 = agree, and 6 = strongly agree. Only respondents with positive responses (options 4–6) are shown in the table. S1: Secondary 1 level; S2: Secondary 2 level; S3: Secondary 3 level.

**Table 2:** Summary of the program implementers' perception towards their own performance.

	Respondents with positive responses (options 4–6)							
	S1		S2		S3		Overall	
	*n*	%	*n*	%	*n*	%	*N*	%
(1) I have a good mastery of the curriculum.	1142	90.21	897	87.34	825	87.77	2864	88.59
(2) I prepared well for the lessons.	1143	90.71	919	89.57	835	89.11	2897	89.89
(3) My teaching skills were good.	1153	91.51	921	89.59	832	88.79	2906	90.11
(4) I have good professional attitudes.	1220	96.60	993	96.69	898	95.63	3111	96.35
(5) I was very involved.	1194	94.46	965	93.87	863	91.81	3022	93.50
(6) I gained a lot during the course of instruction.	1083	86.02	859	83.72	786	83.71	2728	84.62
(7) I cared for the students.	1237	97.63	1002	97.47	913	97.13	3152	97.43
(8) I was ready to offer help to students when needed.	1247	98.42	1010	98.25	919	97.77	3176	98.18
(9) I had much interaction with the students.	1200	94.79	964	93.87	875	93.09	3039	94.00
(10) Overall speaking, I have very positive evaluation of myself as an instructor.	1228	96.92	981	95.43	899	95.64	3108	96.07

*Note: *all items are on a 6-point Likert scale with 1 = strongly disagree, 2 = disagree, 3 = slightly disagree, 4 = slightly agree, 5 = agree, and 6 = strongly agree. Only respondents with positive responses (Options 4–6) are shown in the table. S1: Secondary 1 level; S2: Secondary 2 level; S3: Secondary 3 level.

**Table 3:** Summary of the program implementers' perception towards the program effectiveness.

The extent to which the Tier 1 Program (i.e., the program in which all students have joined) has helped your students	Respondents with positive responses (options 3–5)							
	S1		S2		S3		Overall	
	*n*	%	*n*	%	*n*	%	*N*	%
(1) It has strengthened students' bonding with teachers, classmates, and their families.	1163	91.79	932	90.57	846	90.00	2941	90.88
(2) It has strengthened students' resilience in adverse conditions.	1127	89.02	905	87.78	829	88.19	2936	91.84
(3) It has enhanced students' social competence.	1185	93.53	941	91.63	854	90.95	2541	93.73
(4) It has improved students' ability in handling.	1164	91.94	934	90.77	842	89.77	2108	88.83
(5) It has enhanced students' cognitive competence.	1118	88.38	887	86.03	831	88.50	1964	72.74
(6) Students' ability to resist harmful influences has been improved.	1126	89.08	899	87.37	814	86.69	2004	64.79
(7) It has strengthened students' ability to distinguish between the good and the bad.	1181	93.21	948	91.95	856	91.36	2463	76.37
(8) It has increased students' competence in making sensible and wise choices.	1152	91.07	915	88.92	838	89.15	2938	90.79
(9) It has helped students to have life reflections.	1104	87.48	909	88.34	839	89.26	2917	91.61
(10) It has reinforced students' self-confidence.	1063	83.97	835	81.07	769	81.81	2389	86.31
(11) It has increased students' self- awareness.	1179	93.87	937	90.97	864	91.91	2243	90.52
(12) It has helped students to face the future with a positive attitude.	1091	86.72	884	85.91	818	87.11	1916	74.35
(13) It has helped students to cultivate compassion and care about others.	1109	88.23	903	87.84	823	87.55	1995	63.58
(14) It has encouraged students to care about the community.	1032	82.23	846	82.14	765	81.30	2205	68.46
(15) It has promoted students' sense of responsibility in serving the society.	1030	81.94	843	81.77	779	82.78	2712	84.07
(16) It has enriched the overall development of the students.	1189	94.67	953	92.43	877	93.20	3044	95.27

*Note: *all items are on a 5-point Likert scale with 1 = unhelpful, 2 = not very helpful, 3 = slightly helpful, 4 = helpful, and 5 = very helpful. Only respondents with positive responses (Options 3–5) are shown in the table. S1: Secondary 1 level; S2: Secondary 2 level; S3: Secondary 3 level.

**Table 4:** Means, standard deviations, Cronbach's alphas, and means of interitem correlations among the variables by grade.

	S1		S2		S3		Overall	
	*M*	*α*	*M*	*α*	*M*	*α*	*M*	*α*
	(SD)	(Mean^#^)	(SD)	(Mean^#^)	(SD)	(Mean^#^)	(SD)	(Mean^#^)
Program content (10 items)	4.47 (.42)	.95 (.65)	4.39 (.47)	.95 (.66)	4.36 (.53)	.95 (.67)	4.41 (.48)	.95 (.66)
Program implementers (10 items)	4.68 (.31)	.93 (.59)	4.62 (.36)	.94 (.63)	4.63 (.40)	.96 (.69)	4.65 (.36)	.94 (.64)
Program effectiveness (16 items)	3.44 (.40)	.97 (.71)	3.41 (.43)	.98 (.71)	3.41 (.44)	.98 (.73)	3.42 (.42)	.98 (.71)
Total effectiveness (36 items)	4.07 (.34)	.98 (.52)	4.02 (.39)	.98 (.57)	4.01 (.42)	.98 (.60)	4.04 (.38)	.98 (.56)

^#^Mean interitem correlations.

**Table 5:** Multiple regression analyses predicting program effectiveness.

	Predictors			Model
	Program content	Program implementers		
	*β* ^ a^	*β* ^ a^	*R*	*R* ^2^
S1	.71**	.09	.77	.59
S2	.65**	.20**	.80	.64
S3	.74**	.09	.82	.67
Overall	.70**	.13**	.79	.63

^a^Standardized coefficients.

*P < .05, **P < .01.
